# Field study of compound microbial agents for soil improvement and microbial community dynamics on rocky slopes in Southwest China

**DOI:** 10.1038/s41598-025-17469-1

**Published:** 2025-09-26

**Authors:** Ying Lv, Rongyang Fan, Yinghao Zhao, Hao Chen, Haoyu Wu, Hongjin Huang, Yu Cheng, Xingyu Liu, Mingfan Guo

**Affiliations:** 1 Institute of Earth Science, China University of Geosciences (Beijing), Beijing, 100083 China; 2 China Energy Engineering Group, Guangxi Electric Power Design Institute Co., Ltd., Nanning, 530001 China

**Keywords:** Biological soil crust, Ecosystem functions, Microbial remediation, Soil fertility, Vegetation restoration, Biological techniques, Ecology, Environmental sciences

## Abstract

Engineering construction and mining activities have resulted in numerous exposed rocky slopes, posing significant geological and ecological challenges. To address these issues, this study developed compound microbial agents composed of functional microorganisms and applied them in a field-based ecological restoration project on typical high and steep rocky slopes in Southwest China. The aim was to restore the micro-ecological environment of the slopes by reconstructing the soil conditions. After 240 days of restoration, soil available phosphorus increased from < 2.0 to 6.10 mg/kg, available potassium from 62.80 to 75.00 mg/kg, organic matter from 8.90 to 12.86 g/kg, and organic carbon from 0.70 to 0.73%. Total nitrogen and total phosphorus slightly increased, indicating improved soil fertility. The addition of compound microbial agents enhanced microecological stability while maintaining the overall structure of the indigenous microbial communities. The progressive development of biological soil crusts and rock fissures facilitated the colonization of algae, lichens, mosses, and higher plants. By the 8th month, vegetation coverage exceeded 30% in some areas. This study presents an effective field-based model for the microbial ecological restoration of rocky slopes and offers insights into ecosystem-recovery mechanisms supporting sustainable land management.

## Introduction

With the rapid development of the social economy, the construction of infrastructure such as roads, water, and power supply, as well as open-pit mining activities, has created numerous steep and soil-less rocky slopes. These conditions may lead to significant geological and ecological problems^1,2^. For instance, exposed rocks on the slopes are susceptible to collapses, landslides, and debris flows under the influence of natural factors such as wind, sun, and rain, thereby severely threatening the safety and property of nearby residents. Additionally, biodiversity decreases, landscapes are destroyed, and the visual pollution created is highly discordant with the surrounding natural scenery^3^. Particularly, high and steep rocky slopes present more ecological constraints compared to soil slopes. They lack soil cover as well as the essential edaphic and hydrological conditions required for vegetation establishment. The high and steep slopes have poor substrate attachment conditions. These slopes exhibit pronounced spatial heterogeneity, and the prevailing ecological conditions are highly adverse. Moreover, the geological conditions are complex and unstable^4–6^. In many typical sites, especially those underlain by carbonate or limestone formations, the rock mass exhibits poor weathering properties and limited fissure development. This results in extremely shallow or even absent soil layers, severely limited water-retention capacity, and highly constrained zones for microbial colonization and root penetration. The ecological restoration of such slopes is challenging and represents a key difficulty in ecological management^7–9^. These issues exert substantial constraints on the sustainable socio-economic development of China. Therefore, the ecological restoration and management of these rocky slopes are of great importance.

Since the 1990s, with the development of ecological restoration technologies for slopes under different site conditions, vegetation reconstruction techniques that combine slope protection and greening functions have received increasing attention. Especially with the rapid development of highway construction in China, a large number of practical and theoretical studies on vegetation reconstruction for highway slopes have been carried out by researchers and engineers. They have introduced and absorbed advanced foreign technologies, achieving a series of research results that have essentially solved the vegetation reconstruction problems of soil and soil-rock slopes on Chinese highways^10^. However, for high and steep rocky slopes, existing vegetation reconstruction methods, such as hydroseeding, pit planting, or planting bags, often suffer from poor substrate adhesion, inadequate nutrient supply, and low plant survival under harsh environmental conditions. These methods are also difficult to apply consistently across highly heterogeneous and steep rock surfaces. Furthermore, the lack of weathered matrix or continuous fissure systems on such slopes limits both physical anchoring and nutrient retention, severely constraining the effectiveness of conventional interventions. Most of these technologies focus on physical or chemical stabilization and lack the ability to rebuild ecological functions^11^. Although techniques like spray-mixing with binders or vegetation-growing concrete have been tested, the addition of materials like cement often raises substrate alkalinity, hinders plant growth, and increases maintenance costs, leading to rapid degradation of restoration effects^12–14^. Therefore, the ecological restoration of high and steep rocky slopes remains a critical technical challenge, thereby creating an urgent need for innovative biological approaches capable of reconstructing soil function and sustaining long-term vegetation growth.

Soil microorganisms play a pivotal role in terrestrial ecosystems, particularly in nutrient cycling and soil structural development. Compared with traditional engineering methods, microbial technologies offer advantages such as low cost, sustainability, and ecological compatibility. In recent years, researchers have isolated and applied various beneficial microorganisms for ecological restoration, including nitrogen-fixing bacteria^15^ carbon-fixing bacteria^16^ phosphate- and potassium-solubilizing bacteria^17,18^ and sulfate-reducing bacteria^19^. These microbes can enhance nutrient availability, stimulate biological soil crust formation, and improve overall ecosystem resilience^20–24^. In addition to nutrient cycling, microbial communities—particularly fungi and bacteria—can excrete extracellular polymeric substances (EPS), form mycelial networks, and bind soil particles together, thereby improving soil aggregate stability and increasing erosion resistance^25,26^. Biological soil crusts (biocrusts), formed through the binding of bacteria, fungi, algae, lichens, mosses, and their secretions with soil particles under specific climatic conditions, are considered pioneer communities in extremely degraded ecosystems and play a crucial role in promoting vegetation succession^27,28^. Furthermore, the establishment of vegetation facilitated by microbial processes contributes to erosion control through root reinforcement of shallow soil layers, interception of rainfall, and accumulation of litter that protects the soil surface and supports further microbial colonization^29–31^. However, few studies have systematically integrated these microorganisms into customized compound microbial agents for use on high and steep rocky slopes. More importantly, long-term field-based evaluations of their ecological effects—such as microbial community shifts, soil fertility improvement, and vegetation establishment—are still lacking.

To address these gaps, this study proposes an integrated field-based approach using compound microbial agents constructed from functional microorganisms previously screened in laboratory and pilot experiments. Unlike traditional physical or chemical slope stabilization methods, this approach focuses on restoring ecological functionality through microbial-driven soil formation and nutrient cycling. These agents were applied to a real-world rocky slope site in Southwest China to assess their efficacy in improving soil conditions, supporting vegetation growth, and driving microbial community development. The core scientific question concerns whether microbial agents can not only persist but also perform ecological functions under extreme slope conditions characterized by limited soil and moisture availability. To clarify this, the synergistic effects of nutrient-solubilizing, carbon-fixing, and sulfur-cycling microorganisms within fractured rock systems were investigated. High-throughput sequencing and soil nutrient profiling were employed to examine the interactions among microorganisms, soil substrates, and early-stage vegetation, thereby revealing the dynamic coupling of microbial succession and ecological recovery. The results contribute to a theoretical basis for large-scale restoration of severely degraded slopes and present a novel biotechnological pathway for addressing longstanding ecological limitations in steep rocky environments.

## Materials and methods

### Materials

#### Experimental site

The experimental site is located in the northeastern region of the Guangxi Zhuang Autonomous Region, China (N25˚23′, E110˚21′), at an altitude of approximately 190 m and a slope gradient of about 70°, characterized by a stepped rocky slope. The slope forms part of a former quarry subjected to prolonged anthropogenic excavation, resulting in the near-complete removal of vegetation and soil layers. The original mountain vegetation was well developed, with trees and shrubs mainly consisting of arbor and shrub species. After exploitation, most of the bedrock is exposed, and some areas with well-developed structural fractures appear fragmented.

**Fig. 1:**
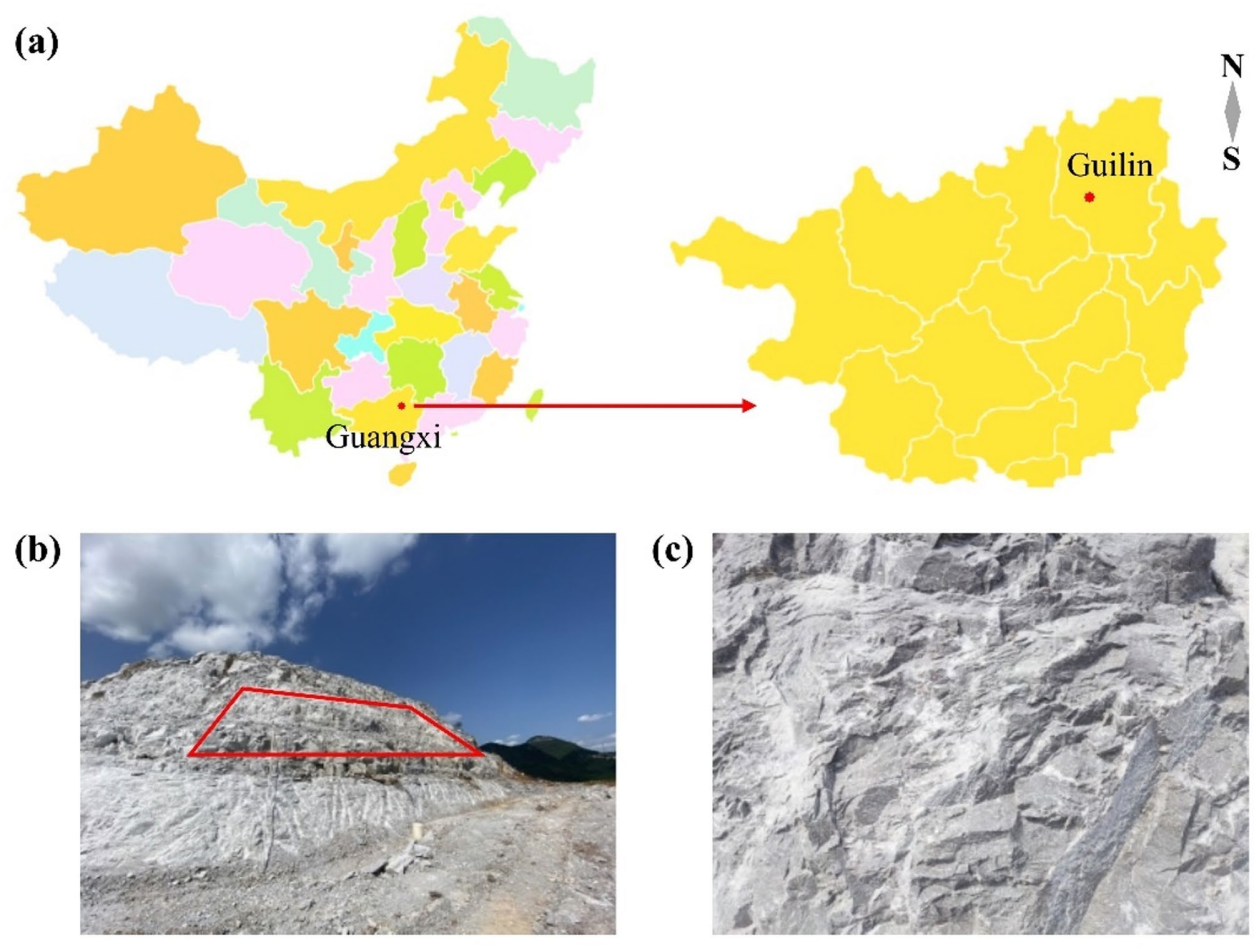
Distribution of the experimental site and overview of the restoration area. (**a**) Geographical location of the experimental site; (**b**) Panoramic view of the experimental site (highlighted in red is the experimental area); (**c**) Partial view of the experimental site.

The mountain is an isolated peak on a plain, with the main rock-soil type being Quaternary carbonate rock. The rock mass consists of medium-thick bedded limestone and flint-bearing limestone. Due to the influence of folds and faults, bedrock exposed on steep mountain peaks has developed fractures, locally forming unstable rocks and collapses. Quarrying activities have markedly altered the natural geomorphology, producing steep, hard rock faces with sparse fissuring and a complete loss of natural soil cover, thereby creating formidable challenges for ecological restoration. Figure 1 shows the area to be restored. As can be seen, the rock formation has high strength, is dense and hard, and the rock mass structure is relatively intact. The rock wall has very few developed fractures and no vegetation cover.

#### Strains

The compound microbial agents were composed of various functional microorganisms previously constructed and preserved in the laboratory. These strains—including *Arthrobacter nitrophenolicus* N18 (deposit number: CCTCC M 20232650), *Bacillus* sp. ZJ-1, *Acinetobacter* sp. P2 (deposit number: CCTCC M 20232652), *Pseudomonas* sp. LY-13 (deposit number: CCTCC M 2022520), and *Desulfovibrio vulgaris* EM2 (deposit number: CCTCC M 2017645)—were originally isolated from ecologically similar karst and rocky environments and systematically screened and maintained through prior laboratory and pilot-scale experiments^32,33^. Their selection was based on proven performance under conditions mimicking karst rocky slopes, including high pH, low nutrient availability, and limited water retention. Specifically, *Arthrobacter* and *Bacillus* are known for their strong environmental adaptability and plant growth-promoting capabilities through the production of hormones and siderophores; *Acinetobacter* and *Pseudomonas* exhibit high efficiency in phosphorus and potassium solubilization as well as cellulose degradation; *Desulfovibrio* contributes to sulfur cycling and microenvironment regulation.

Together, these microorganisms demonstrate synergistic functions in nutrient mobilization, soil microstructure improvement, and enhancement of plant–microbe interactions on fragmented, nutrient-poor rock surfaces. Functional validation from pre-experiments confirmed their ability to solubilize phosphorus and potassium, produce vegetation growth hormones, degrade cellulose, secrete siderophores, and supply essential sulfur elements—highlighting their superior suitability for microbial restoration in high and steep rocky slope environments.

#### Medium

Two types of media were used for different cultivation purposes.

Tryptic soy broth (TSB) medium: The medium consisted of 17.0 g/L tryptone, 3.0 g/L soytone, 2.5 g/L K_2_HPO_4_, 5.0 g/L NaCl, and 2.5 g/L glucose. The pH was adjusted to 7.0, and the medium was sterilized at 121 °C for 20 min before use. This medium was primarily used for the initial activation and enrichment of laboratory-preserved strains due to its rich nutrient composition, which supported robust bacterial recovery and early growth.

Medium for scale-up culture: The medium included 1.0 g/L yeast extract, 0.2 g/L glucose, 0.5 g/L K_2_HPO_4_, 1.0 g/L NaCl, 1.0 g/L MgSO_4_, and 1 mL of 60% sodium lactate solution. This formulation was used for stepwise large-volume expansion of the bacterial cultures, with a composition optimized to simulate the field environment while maintaining microbial viability and functional expression. Each component was proportionally added to 5 L, 50 L, and 2000 L water storage tanks (made of polyethylene) for stepwise scale-up culture). After adding clean water, the mixture was thoroughly dissolved before use.

### Methods

#### Scale-up culture of compound microbial agents

The compound microbial agents were cultured using a stepwise scale-up culture method. The first scale-up culture was conducted in the laboratory: five preserved strains were each inoculated into 300 mL of sterile TSB liquid medium and cultured on a shaker at 30 °C and 160 r/min. When the OD_600_ of the bacterial suspension reached 1.0, the cultures were centrifuged at 4 °C and 8000 r/min for 30 min to collect the bacterial cells. Then the five microbial cell suspensions were mixed in equal proportions and resuspended in a new 500 mL sterile TSB liquid medium, which was then used as the seed liquid for compound microbial agents at the experimental site.

**Fig. 2:**
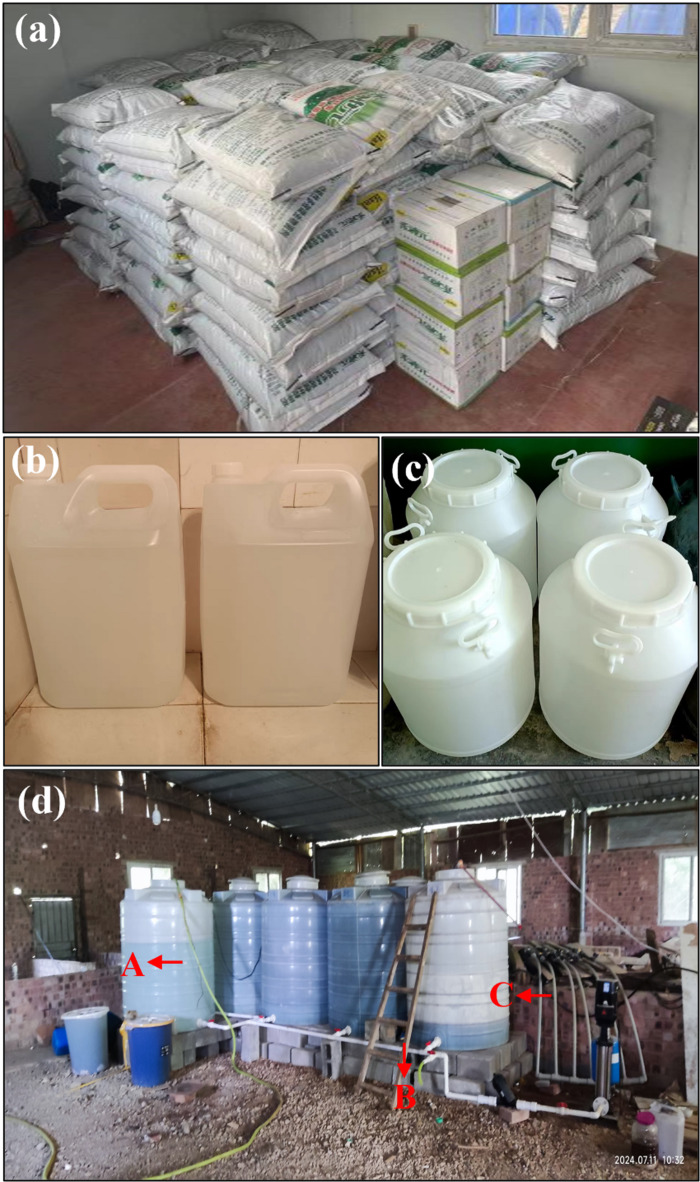
On-site scale-up culture experiment record diagram. (**a**) Medium component; (**b**) 5 L scale-up system; (**c**) 50 L scale-up system; (**d**) 2000 L scale-up system (A: water inlet; B: water outlet; C: water supply pump).

The experimental site was equipped with multiple 5 L, 50 L, and 2000 L water storage tanks (made of polyethylene). Based on the volume of the storage tanks, the scale-up culture medium was added, filled with clean water, and stirred to dissolve the medium fully, thus serving as the microbial culture apparatus. The second scale-up culture was conducted in the 5 L culture tanks. After culturing for 7–14 days (the culture period was determined based on microbial growth conditions), the culture liquid was transferred to the 50 L culture tanks for the third scale-up culture. Subsequently, the culture was gradually scaled up in the 2000 L culture tanks with an inoculation ratio of 5%-10%. When the OD_600_ of the compound microbial agents in the 2000 L culture tanks reached approximately 0.5, the culture could be used for on-site microbial spraying. After each spraying, at least 1/10 of the culture liquid remaining in the tank could be used as seed liquid. The liquid culture medium was replenished for repeated scale-up culture. The on-site scale-up culture experiment records are shown in Fig. 2. Periodically, culture liquid was collected from the culture tanks for microbial high-throughput sequencing analysis to reveal the changes in microbial community structure and diversity within the compound microbial agents.

#### On-site spraying of compound microbial agents

The on-site spraying of compound microbial agents started in September 2023 and continued until April 2024. Since the culture tanks were located below the rocky slope, the spraying apparatus needed to be set up in advance. To prevent the agents from clogging the spray pipes and to lower the temperature of rock wall (which reached 60–70 °C in the experimental area in September), it was necessary to spray clean water for 30 s before each application of the compound microbial agents. The experimental area covers a surface area of approximately 600 m^2^with an initial total daily spray volume of about 0.844 m^3^. The spraying frequency was divided into three stages based on the restoration timeline: (1) Initial Restoration Stage (September 2023 to December 2023): The experimental area was sprayed five times daily at 8 p.m., 11 p.m., 2 a.m., 5 a.m., and 8 a.m., with each spraying lasting 3 min. Additionally, clean water was sprayed during the day to maintain temperature and humidity, with eight sprays daily at 9 a.m., 11 a.m., 12 p.m., 1 p.m., 2 p.m., 3 p.m., 4 p.m., and 6 p.m. (2) Winter Period (December 2023 to February 2024): Spraying was maintained only in specific areas, with two sprays daily at 11 a.m. and 4 p.m., each lasting 4 min. During this period, to reduce microbial dormancy and physical loss caused by freezing, the experimental rock slope was partially covered with geotextile and plastic film. These coverings helped maintain surface temperature and moisture conditions during cold weather, thereby supporting the survival and stability of developing microbial and plant communities. The coverings were removed in early spring prior to the resumption of full-area spraying. (3) Final Restoration Stage (March 2024 to April 2024): The entire area was sprayed twice daily at 6 a.m. and 8 p.m., with each spray lasting 4 min, using approximately half the initial volume.

The rationale behind this spraying schedule was derived from preliminary field tests, which showed high evaporation and poor water-holding capacity on the rocky surface, especially during the hot season. Intensive spraying in the initial stage ensured adequate microbial colonization and survival in fractured rock systems, where fluid agents could easily run off. Gradual reduction in spraying frequency in the later stages simulated natural succession and microbial self-regulation while improving operational efficiency and conserving water resources. This staged spraying strategy was designed to balance initial microbial activation with long-term ecological sustainability.

#### Restoration effectiveness in the experimental area of high and steep rocky slope

A 240-day ecological restoration project monitoring was conducted at the experimental site. During the monitoring period, the growth of vegetation in the experimental area was continuously recorded. During the monitoring period, the growth of vegetation in the experimental area was continuously recorded through visual inspection, standardized photographic documentation, and manual measurements of vegetation cover percentage. Specifically, vegetation cover was estimated using 1 m × 1 m fixed quadrats at representative locations, and photographs were taken at regular intervals to track the development of plant coverage and species composition over time. This was done to evaluate the effectiveness of the compound microbial agents in restoring typical high and steep rocky slopes. Additionally, soil samples were collected from developed cracks at four time points: days 15 (representing the early phase following microbial agent application, primarily aimed at verifying microbial retention and initial activity), 60 (corresponding to the initial establishment of vegetation), 210 (capturing the spring peak of biological activity and system stabilization), and 240 (serving as the final evaluation point at the conclusion of the restoration cycle), following the sampling methods specified in “GB/T 36197 − 2018 Soil Quality - Soil Sampling Technical Guidelines”. The samples were transported to the laboratory under low-temperature conditions for subsequent processing. Importantly, due to the extremely limited soil volume available during the early phase of restoration, the amount of sample retrievable on day 15 was only sufficient for microbial community analysis and did not meet the minimum requirements for parallel nutrient testing. Moreover, the combination of the high slope gradient, fragmented and discontinuous soil patches, rapid surface runoff, and significant safety risks made it impossible to designate a physically isolated, untreated control plot on the same slope. While the treated area could be accessed through targeted spraying along a secured path, establishing a control plot would have required leaving adjacent sections entirely untouched, which was unfeasible because any such area would either (1) receive runoff containing microbial agents from upslope, causing cross-contamination, or (2) be located in inaccessible cliff sections where safe sampling and monitoring could not be performed. Therefore, unlike the treatment area, a viable control area with comparable topography, soil retention, and accessibility could not be secured at this site. To address this limitation, a phased sampling strategy was employed. The 15-day sample served as a baseline for microbial community analysis, as sequencing at this stage showed no detectable colonization of the introduced strains. The 60-day sample was selected as an approximate nutrient reference point, as microbial agents had not yet fully exerted their effects and vegetation was only beginning to establish. The 210-day and 240-day samples captured peak and late-stage ecological responses, respectively, enabling the construction of a temporal gradient to infer treatment effects.

Due to the challenging karst terrain and steep rocky conditions, soil sampling was extremely difficult. The site was characterized by a limited surface area and a small number of rock fissures capable of retaining soil. As such, conventional multi-point or composite sampling methods could not be adopted. Instead, a set of fixed sampling sites with relatively large and stable fissures was established at the beginning of the experiment, and soil was repeatedly collected from these same sites throughout the study to ensure consistency. To avoid cross-contamination, each sampling point was clearly marked and physically isolated by natural stone barriers. Sampling tools were thoroughly sterilized before each use, and sample containers were uniquely labeled and sealed immediately after collection. Sampling was consistently conducted from upslope to downslope to minimize the risk of downward runoff contamination. Moreover, it should be emphasized that the soil retained within the fissures primarily originates from the native slope substrate, formed through natural weathering processes and sediment accumulation within karst micro-depressions. Throughout the restoration process, no artificial soil substrates or exogenous additives were introduced to the slope surface. Although the potential influence of airborne dust deposition cannot be entirely excluded, sampling was intentionally confined to deep, stable fissures to minimize external interference and improve data reliability. Due to spatial limitations and variable soil retention across fissures, the amount of soil collected per sampling event varied, but generally ranged around 100 g per site, which was sufficient for subsequent nutrient and microbial analyses.

Each collected soil sample was pre-treated and divided into two portions. One portion was used for soil nutrient analysis, including indicators such as total carbon (TC), total nitrogen (TN), total phosphorus (TP), total potassium (TK), organic carbon (OC), organic matter (OM), available nitrogen (AN), available phosphorus (AP), and available potassium (AK). The second portion was allocated for microbial community structure analysis. Based on preliminary trials that showed considerable variability in nutrient measurements, three technical replicates were included in the nutrient analysis to improve statistical reliability. However, for microbial diversity analysis, only two replicates could be processed due to the limited volume of soil available from each fissure. In addition, a portion of the soil had to be reserved to ensure plant establishment and ecological integrity, which further constrained the number of microbial replicates. In addition, soil nutrient content analysis was conducted with technical support from the National Institute of Metrology, China, and Scientific Compass, while microbial community structure analysis was carried out with the assistance of Majorbio Bio-Pharm Technology Co., Ltd.

Furthermore, long-term field monitoring revealed that various biological soil crusts—such as algae, lichens, and mosses—gradually developed on the rock surface. Representative crust samples were collected from fixed observation sites using sterile tools, with each sample covering an area of approximately 2 cm × 2 cm. The collected materials were carefully transferred into sterile containers, stored in ice boxes, and promptly transported to the laboratory for microbial community analysis. The procedures for microbial DNA extraction, sequencing, and diversity index calculation were consistent with those applied to the soil samples.

### Measurements

The measurement of total carbon and organic carbon in the samples was performed in accordance with “HJ 695–2014 Soil - Determination of organic carbon - Combustion oxidation nondispersive infrared absorption method”. The measurement of total nitrogen followed “HJ 717–2014 Soil quality - Determination of total nitrogen - Modified Kjeldahl method”. The measurement of total phosphorus was based on “HJ 632–2011 Soil - Determination of Total Phosphorus by alkali fusion-Mo-Sb Anti spectrophotometric method”. Total potassium content was determined using EPA 6010D (2018). Organic matter content was measured according to “NY/T 1121.6–2006 Soil Testing - Part 6: Method for determination of soil organic matter”. The measurement of available nitrogen was based on the alkaline hydrolysis distillation method. The measurement of available phosphorus referred to “HJ 704–2014 Soil quality - Determination of available phosphorus-Sodium hydrogen carbonate solution-Mo-Sb anti spectrophotometric method”. The measurement of available potassium referred to “NY/T 889–2004 Determination of exchangeable potassium and non-exchangeable potassium content in soil”.

All soil samples were collected from fissures at a depth of 0–15 cm using sterilized stainless steel scoops to avoid surface contamination. The samples were air-dried and sieved (2 mm) before chemical analyses. Nutrient measurements were performed using standard laboratory equipment, including an Elementar vario TOC cube (for TC and OC), a Kjeltec 8400 Analyzer Unit (for TN), a PerkinElmer Lambda 35 UV-Vis Spectrophotometer (for TP and AP), and an Agilent 5110 ICP-OES (for TK and AK).

The microbial community structure was analyzed and visualized using the MagicGene Cloud Platform (http://cloud.magigene.com). Alpha diversity indices, including Shannon and Chao1, were calculated using QIIME2 (version 2023.2) and the “vegan” package in R software (R Core Team, version 4.2.2; https://www.r-project.org/)^34,35^. Microbial DNA was extracted from 0.5 g of fresh soil using the FastDNA Spin Kit for Soil (MP Biomedicals, USA), and the V3–V4 region of the bacterial 16 S rRNA gene was amplified using universal primers 341 F/806R. PCR products were sequenced on the Illumina MiSeq platform. The sequences obtained were assigned to taxonomic ranks using a reference database, and the relative abundance of major phyla and dominant genera was analyzed. Temporal variations in microbial community composition were illustrated using stacked bar plots and heatmaps to reveal succession trends over the four sampling time points. Principal taxa associated with different restoration stages were further discussed based on their ecological functions and relative abundance changes.

Soil nutrient data were organized with Microsoft Excel 2022, and statistical graphs were generated using Origin 2017. All results are expressed as mean ± standard error (SE). Each measurement was conducted in triplicate. Significance tests were performed using one-way ANOVA and Tukey’s post hoc test, with p-values less than 0.05 considered statistically significant.

## Results and discussion

### Changes in soil fertility at the experimental site

High and steep rocky slopes are characterized by shallow soil layers, well-developed binary structures, and mismatched water and soil resources, making the ecological environment extremely fragile. Therefore, the soil ecosystem in such areas is a crucial foundation for vegetation restoration, and improving soil quality is one of the main technical measures to enhance the effectiveness of ecological restoration on high and steep rocky slopes^36^. Based on this, studying the impact and mechanisms of different soil improvement measures on soil quality has become a hot topic in vegetation restoration ecology of such sites. Table 1 lists the changes in nutrient content in soil samples from the experimental area during different restoration periods. All nutrient values were expressed as mean ± standard error (SE) based on three independent replicates. One-way ANOVA was used to compare nutrient contents across different restoration periods, followed by Tukey’s post hoc test for pairwise comparisons. Statistical significance is indicated by asterisks: *p* < 0.05 (*), *p* < 0.01 (**). These statistical analyses were conducted using Origin 2017 software.

At the 60-day restoration stage, the content of total carbon, total nitrogen, total phosphorus, and total potassium in the soil was 9.55%, 636.00 mg/kg, 121.00 mg/kg, and 3140 mg/kg, respectively. The content of available nitrogen and available potassium was 85.70 mg/kg and 62.80 mg/kg, respectively, while the content of available phosphorus was below 2.0 mg/kg. High and steep rocky slopes generally have nutrient element limitations, one reason being the risk of soil and water erosion in these areas, which leads to a reduction in nutrient content^37^. Additionally, the content of organic carbon and organic matter was only 0.70% and 8.90 g/kg. This indicates that the soil in this area is poor and lacks organic matter, unable to support basic vegetation growth.

**Table 1:** Nutrient content in soil samples from the experimental area at different restoration periods.

Restoration period	TC (%)	TN (mg/kg)	TP (mg/kg)	TK (mg/kg)	AN (mg/kg)
60 d	9.55 ± 0.46^*^	636.00 ± 32.15	121.00 ± 11.04^**^	3140 ± 32.24^**^	85.70 ± 12.38^**^
210 d	9.75 ± 0.89^*^	649.00 ± 44.75	239.00 ± 6.82^**^	2980 ± 23.96^**^	33.63 ± 6.85^*^
240 d	9.59 ± 0.23^*^	641.30 ± 56.92	149.00 ± 13.27^**^	3120 ± 18.79^**^	27.00 ± 2.75^*^

Restoration period	AP (mg/kg)	AK (mg/kg)	OC (%)	OM (g/kg)	
60 d	< 2.0^**^	62.80 ± 12.02^*^	0.70 ± 0.03^*^	8.90 ± 0.75^**^	
210 d	11.10 ± 0.52^**^	59.80 ± 8.46^**^	1.06 ± 0.12^**^	18.90 ± 1.39^**^	
240 d	6.10 ± 1.33^**^	75.00 ± 10.27^**^	0.73 ± 0.09^*^	12.86 ± 3.17^**^	

Carbon, nitrogen, phosphorus, and potassium are fundamental macronutrients that underpin vegetation establishment and are thus pivotal to the success of ecological restoration initiatives. The geochemical and eco-physiological consequences of their deficiency warrant in-depth investigation, as they form the scientific basis for enhancing ecological restoration quality. In this study, when the restoration period reached 210 days, compared to the nutrient levels measured at day 60, the contents of total carbon, total nitrogen, total phosphorus, available phosphorus, organic carbon, and organic matter in the soil of the experimental area significantly increased (*p* < 0.05 or *p* < 0.01). Among them, total phosphorus, available phosphorus, organic carbon, and organic matter showed highly significant increases (*p* < 0.01), while total carbon and total nitrogen increased significantly (*p* < 0.05). The contents of total potassium and available potassium slightly decreased, and only the content of available nitrogen showed a more noticeable decline. When the restoration period was extended to 240 days, except for increases in the contents of total potassium and available potassium, the test results for all other indicators were lower than those at the 210-day restoration period. However, compared to the 60-day restoration stage, there was no significant change in total potassium content, the content of available nitrogen continued to decrease (*p* < 0.05), and the contents of all other indicators significantly increased. Specifically, the content of total carbon increased by 0.04%, total nitrogen and total phosphorus increased by 5.3 mg/kg and 28 mg/kg, respectively; available phosphorus increased from below the detection limit to 6.10 mg/kg, showing a highly significant increase (*p* < 0.01); available potassium increased to 75.00 mg/kg, corresponding to an increase of 19.43%; organic carbon increased from 0.70 to 0.73%, and organic matter increased from 8.90 g/kg at day 60 to 12.86 g/kg at day 240, which represented a highly significant change (*p* < 0.01).

Compared to previous studies conducted in karst or rocky fissure environments, the improvements in organic matter and available phosphorus observed in this study are notably superior. For example, Tan et al. reported only a 10–20% increase in organic matter content after vegetation-based restoration interventions in similar southwestern karst regions^38^. Lin et al. found that even in developed karst fissure habitats, the available phosphorus content seldom exceeded 4 mg/kg^39^. Moreover, Meng et al. showed that despite reaching an advanced restoration stage, organic carbon and available phosphorus remained at approximately 12.2 g/kg and 3.94 mg/kg, respectively^40^. In contrast, the present study achieved a 44.49% increase in organic matter and an increase in available phosphorus from undetectable levels to 6.10 mg/kg. These findings underscore the efficacy of compound microbial agents in expediting nutrient cycling and soil fertility restoration, particularly within nutrient-deficient, fractured rocky substrates.

Since the microbial agent solution contains some unused nutrients that enter the site with the spraying, the increase in the content of total carbon, nitrogen, phosphorus, and organic matter cannot fully reflect the role of functional microorganisms. The increase in available phosphorus and available potassium content, to some extent, indicates an enhancement in the nutrition cycling activity at the site, with the growth and metabolic activities of the compound microbial agents playing a positive role in this process. It should be noted that the fluctuation of nutrient levels over time may not be solely attributed to nutrient consumption by vegetation. Exogenous environmental drivers also exert a significant influence. For instance, intense rainfall events can lead to leaching and runoff, washing away nutrients from the shallow rock fissures where soil accumulates. Additionally, as microbial populations grow, competition among microbial species for limited nutrients may intensify, altering nutrient availability and uptake dynamics. Moreover, the specific geomorphic structure of the slope, including uneven fissure development and heterogeneous water retention, may also result in spatial heterogeneity and temporal instability in nutrient accumulation. Collectively, these factors likely account for the observed non-linear temporal trends in soil fertility indicators. Furthermore, although the medium used for culturing the compound microbial agents contains some basic nutrients, its concentration is relatively low and insufficient to directly improve long-term soil fertility. The observed nutrient accumulation is more likely attributed to the metabolic activities of the functional microorganisms, such as nitrogen fixation, phosphorus solubilization, and carbon transformation. These microbial processes enhance nutrient bioavailability and contribute significantly to fertility restoration in nutrient-deficient rocky substrates.

However, overall, the content of various nutrients showed an upward trend compared to the 60-day restoration stage. Notably, organic matter, as an important component of soil, plays a crucial role in supporting vegetation growth, maintaining soil fertility, and improving soil physicochemical properties^41^. In this study, besides the nutrients brought in by the compound microbial agents, soil fertility continued to show an upward trend under the influence of functional microorganisms, reflecting their sustained effectiveness to some extent. The marked increase in organic matter is attributable both to autotrophic microorganisms fixing atmospheric carbon and to the secretion of extracellular polymers during microbial metabolism, which collectively enhance soil fertility.

### Microbial community diversity analysis

**Fig. 3:**
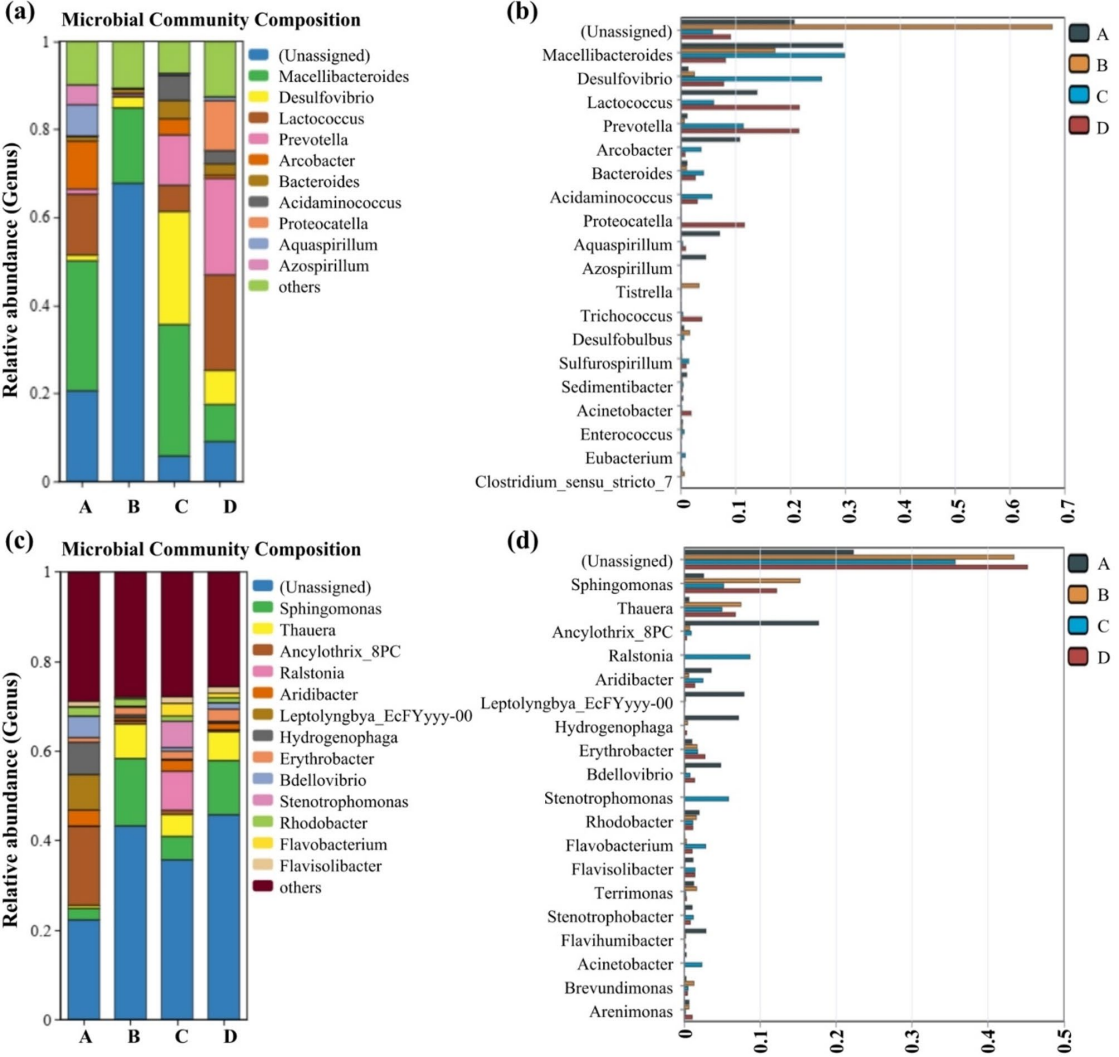
Trends in the microbial community structure over different culture/restoration periods, and the bar chart of the difference test of the most abundant strains (Top 20). (**a**) Microbial liquid during scale-up culture process (A: culture period of 15 days; B: culture period of 60 days; C: culture period of 210 days; D: culture period of 240 days); (**b**) Bar chart of the difference test of the most abundant strains (Top 20) in the microbial liquid during scale-up culture process (A: culture period of 15 days; B: culture period of 60 days; C: culture period of 210 days; D: culture period of 240 days); (**c**) Soil samples (A: restoration period of 15 days; B: restoration period of 60 days; C: restoration period of 210 days; D: restoration period of 240 days); (**d**) Bar chart of the difference test of the most abundant strains (Top 20) in soil samples (A: culture period of 15 days; B: culture period of 60 days; C: culture period of 210 days; D: culture period of 240 days).

The community structure of the microbial agents (collected in the scale-up stage) and the soil microorganisms (collected in the experimental area) under different restoration periods were analyzed, and the results are shown in Fig. 3. It can be seen from Fig. 3a that there are significant differences in the relative abundance of dominant microorganisms in samples collected from different restoration periods. At 15 days of culture, the three most abundant microorganisms in the microbial liquid were *Macellibacteroides* (29.5%), *Lactococcus* (13.8%), and *Arcobacter* (10.9%). At 60 days of culture, the three most abundant microorganisms were *Macellibacteroides* (17.1%), *Desulfovibrio* (2.5%), and *Bacteroides* (1.1%). At 210 days of culture, the three most abundant microorganisms were *Macellibacteroides* (29.8%), *Desulfovibrio* (25.6%), and *Prevotella* (11.4%). When the culture period was extended to 240 days, the three most abundant microorganisms were *Prevotella* (21.9%), *Lactococcus* (21.7%), and *Proteocatella* (11.3%). The difference test of the most abundant strains (Top 20) also demonstrates this point (Fig. 3b), showing significant changes in the main microbial species. However, microorganisms with environmental restoration functions, such as *Desulfovibrio* and *Acinetobacter*, were detected at different times, proving that functional microorganisms can continuously persist during the culture process. On the other hand, *Arthrobacter*, *Bacillus*, and *Pseudomonas*, contained in the compound microbial agents, were not the dominant groups. This may be because the water source used for the culture medium came from complex underground water, where the contained microbial species occupied dominant positions.

Among the widely detected microorganisms, *Macellibacteroides* and *Lactococcus* are common environmental microbes, likely originating from underground water. Additionally, the microbial agents contained some functional microorganisms, including nitrogen-fixing bacteria (*Spirillum*) and sulfate-reducing bacteria (*Desulfovibrio*). Nitrogen-fixing bacteria can convert atmospheric N_2_ into forms of nitrogen that can be absorbed and utilized by vegetation through their metabolic processes, thereby enhancing soil fertility and supporting vegetation growth. Sulfate-reducing bacteria have the ability to promote environmental sulfur cycling by reducing sulfates in the soil. Sulfur is also an essential element for vegetation growth.

As shown in Fig. 3c, the microbial community structure in the site underwent significant changes as the restoration progressed. After 15 days of restoration, the top three microorganisms in terms of abundance were *Ancylothrix* (17.7%), *Leptolynbya* (7.9%), and *Hydrogenophaga* (7.2%). *Ancylothrix* are a group of prokaryotic microorganisms capable of photosynthesis. Some species coexist with bacteria, mosses, ferns, and gymnosperms, and some can even penetrate calcareous rocks or shells (e.g., endolithic algae) or deep soil layers, accelerating the weathering process of rock structures. After 60 days of restoration, the top three microorganisms in the soil were *Sphingomonas* (15.0%), *Thauera* (7.7%), and *Erythrobacter* (1.7%). After 210 days of restoration, the top three microorganisms were *Ralstonia* (8.7%), *Stenotrophomonas* (5.9%), and *Sphingomonas* (5.2%). After 240 days of restoration, the top three microorganisms were *Sphingomonas* (12.1%), *Thauera* (6.5%), and *Erythrobacter* (2.7%). Simultaneously, the content of unclassified microorganisms increased, indicating to some extent that the soil microbial ecosystem was restored and microbial diversity was enhanced. From the difference test of the most abundant strains (Top 20), it can be seen that the dominant microbial species in the soil samples collected at different times changed significantly (Fig. 3d). Indigenous microorganisms like *Ancylothrix* and *Leptolynbya* gradually lost their dominant positions. Given that *Ancylothrix* typically thrive in aquatic environments, their decline in abundance suggests a substantial shift in soil conditions toward those favoring terrestrial microorganisms, indicative of a reconstructed basal microbial community structure. These community shifts may be attributed to multiple ecological factors, including variations in soil moisture and substrate availability. For instance, *Ancylothrix* and *Leptolyngbya* are typically favored in wetter microenvironments, which were prevalent in the early stages of restoration due to residual spray and microbial liquid. As the slope stabilized and biocrust formation enhanced water retention and surface drying, more drought-tolerant and heterotrophic genera like *Sphingomonas* and *Thauera* gained a competitive advantage. In addition, the accumulation of organic matter from microbial metabolism and plant inputs created new nutrient niches that further shaped the succession trajectory.

**Fig. 4:**
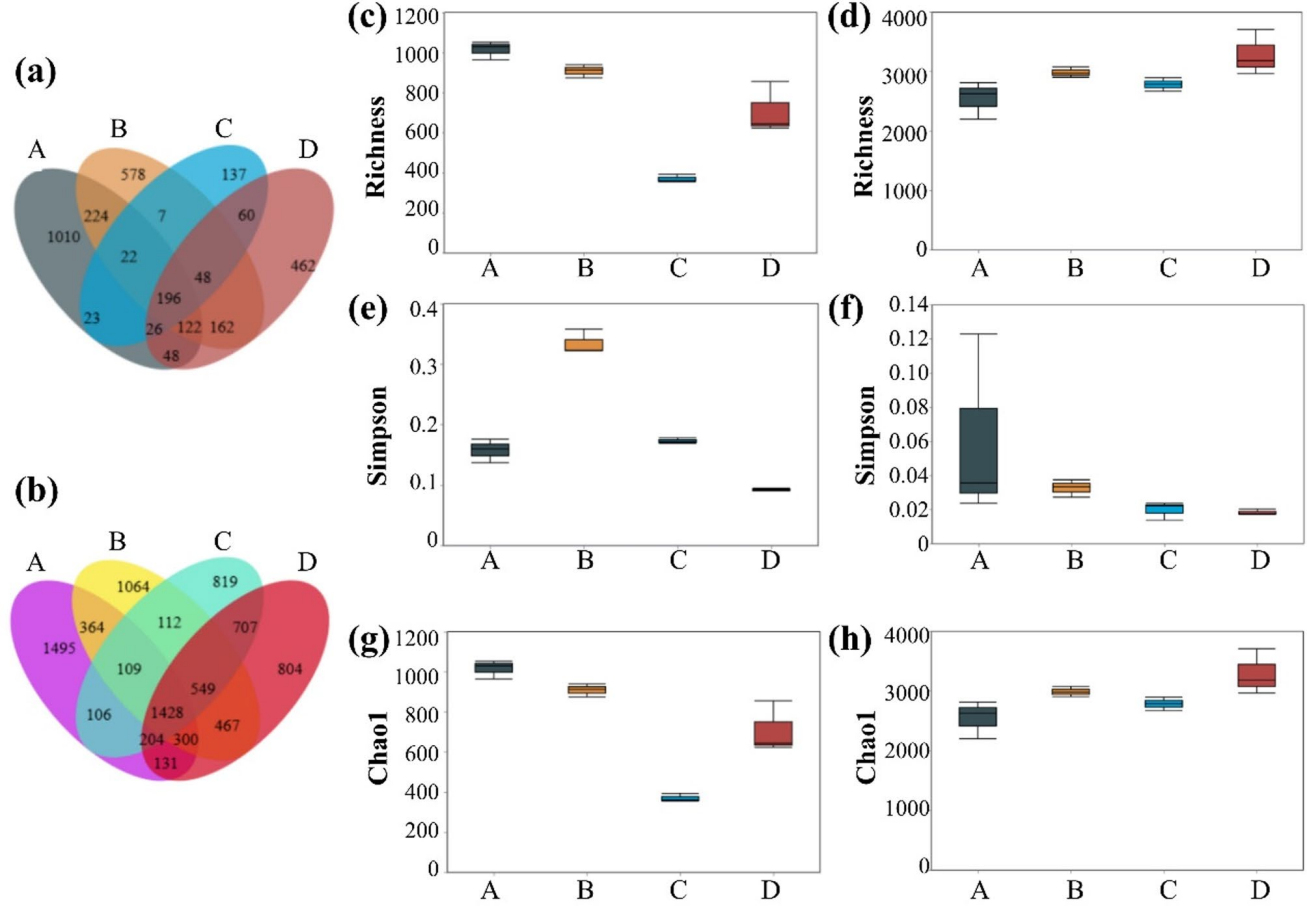
Changes in OTU richness, Richness index, Simpson index, and Chao1 index for microbial liquid (**a**, **c**, **e**, **g**) and soil samples (**b**, **d**, **f**, **h**) at different culture/restoration periods. A: culture period of 15 days; B: culture period of 60 days; C: culture period of 210 days; D: culture period of 240 days.

Additionally, this study also analyzed the microbial diversity indices for the same culture/restoration periods, as shown in Fig. 4. It can be seen that the microbial richness and diversity in the microbial liquid significantly decreased at the 210-day restoration period (during winter). The likely reason is the reduced microbial activity due to lower external environmental temperatures. However, as the temperature increased (entering spring), both microbial diversity and richness showed a substantial recovery. This indicates that temperature is one of the critical influencing factors in the microbial liquid culture process. To maintain the activity and functionality of the microbial liquid, it is necessary to keep the microbial agents in a suitable environment. One of the functional microorganisms in the compound microbial agents, *Desulfovibrio*, was still detectable and remained one of the dominant species in Group C. This indicates that the added functional microorganisms have good tolerance to adverse environments and reflects the potential of the compound microbial agents for environmental restoration. Similarly, for the soil samples, the fluctuations in diversity indices such as Chao1 and Simpson may also be influenced by seasonal variations in temperature and rainfall, which affect microbial metabolic rates, nutrient mobility, and plant–microbe interactions.

In the soil samples from the restoration area, the common and unique OTU numbers show that as the restoration period progresses, the microbial diversity in the site increases. Combining the Chao1 index, it is evident that the number of microbial species shows an upward trend, indicating that the addition of compound microbial agents greatly stimulated the growth of indigenous microorganisms. Richness index analysis revealed that with the progression of the restoration period, the index shows an upward trend, indicating an increase in the total microbial quantity in the site. The gradual decrease in the Simpson index suggests that the microbial community structure in the site is becoming more stable, and the microbial growth conditions are more suitable. In summary, the basal microbial community structure in the site has greatly recovered, and the micro-ecological environment of the slopes has also improved, enhancing the ability to adapt to adverse environments.

**Fig. 5:**
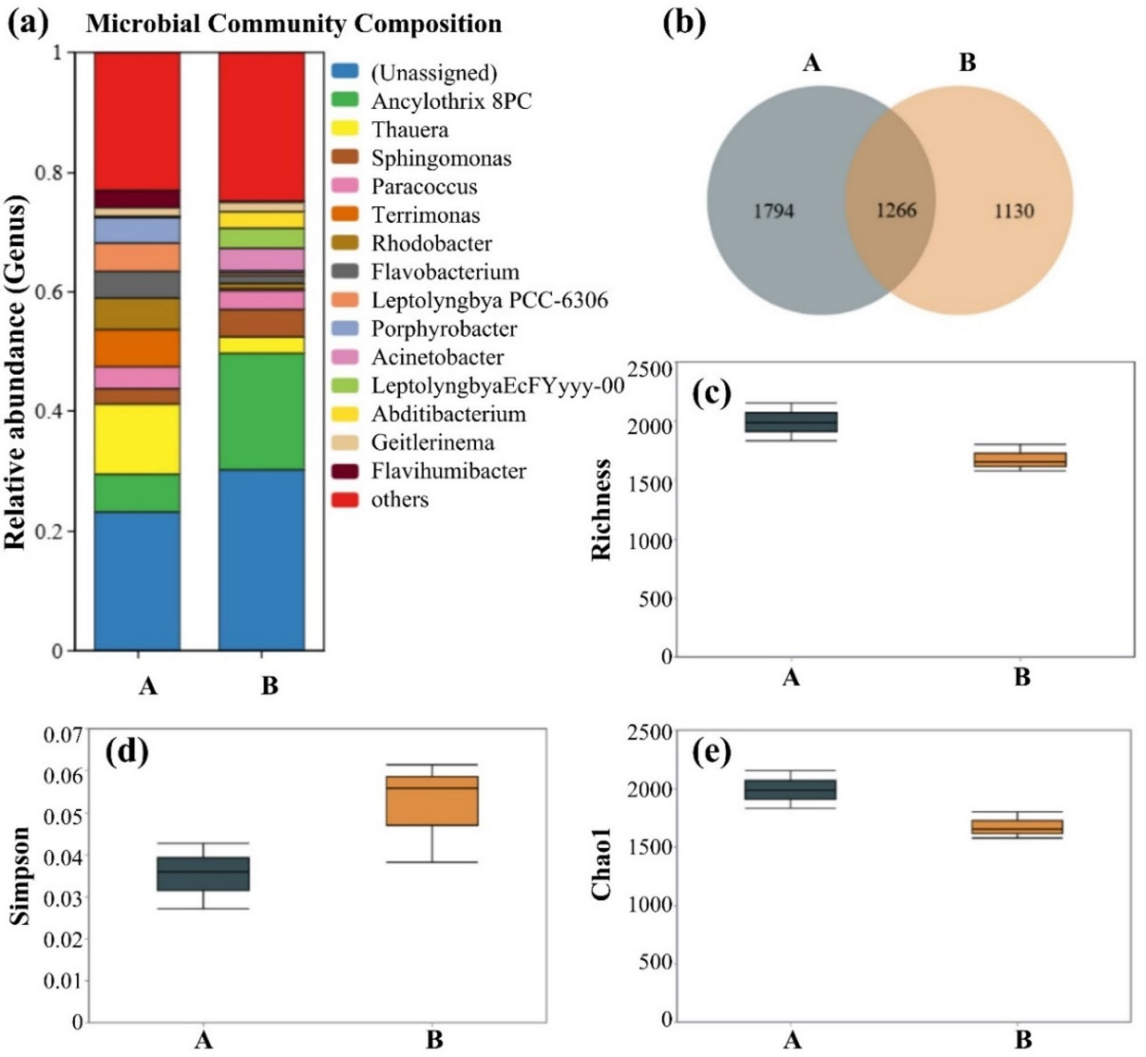
Analysis of bacterial diversity in biological crust structures. (**a**) Microbial community structure; (**b**) Common and unique OTU statistical analysis; (**c**) Box plot of Richness index difference analysis; (**d**) Box plot of Simpson index difference analysis; (**e**) Box plot of Chao1 index difference analysis. A: culture period of 60 days; B: culture period of 210 days.

Additionally, during the continuous monitoring of the high and steep rocky slope, it was observed that as the restoration process progressed, many biological crust structures, such as algae, lichens and mosses, appeared on the rock surface. Samples from these biological crusts were collected and analyzed for microbial diversity, and the results are shown in Fig. 5. In the early stage of restoration, the biological crusts contained high levels of the following microorganisms: *Thauera* (11.5%), *Ancylothrix* (17.8%), *Terrimonas* (6.0%), and *Leptolyngbya* (4.6%). The functional microorganism, *Acinetobacter*, which was artificially added, was also detected, while other functional microorganisms did not occupy a dominant position in the local microbial community due to their lower content. Combining the microbial community structure analysis results of Group B, it can be seen that as the restoration period extended, the main composition of microorganisms in the biological crusts did not change significantly, but the proportions of some microorganisms changed markedly. The content of *Ancylothrix* increased significantly, rising from 6% to about 20%, while *Thauera* decreased from 11 to 3%. The content of other microbial species remained relatively stable. These changes may reflect the influence of microhabitat development on microbial succession within the biocrust. For instance, *Ancylothrix* may benefit from increased sunlight exposure and surface moisture, while the reduction in *Thauera* may be due to reduced availability of organic substrates or increased oxygenation.

As shown in Fig. 5b, the common OTUs between groups A and B are 1266, while the unique OTUs are 1794 and 1130, respectively. The results indicate that the bioremediation process altered the composition of in-situ soil bacteria at the OTU level, leading to significant changes in the bacterial community. This further confirms that the addition of exogenous compound microbial agents changed the soil environment, resulting in changes in the soil microbial community composition. Combined with the changes in the Richness, Simpson, and Chao1 indices, it was found that microbial diversity decreased. This indicates a reduction in the types of microorganisms in the biological crusts and a stabilization of the microbial community structure, with some microorganisms being eliminated due to their inability to adapt to the new environment.

### Vegetation restoration at the experimental site

**Fig. 6:**
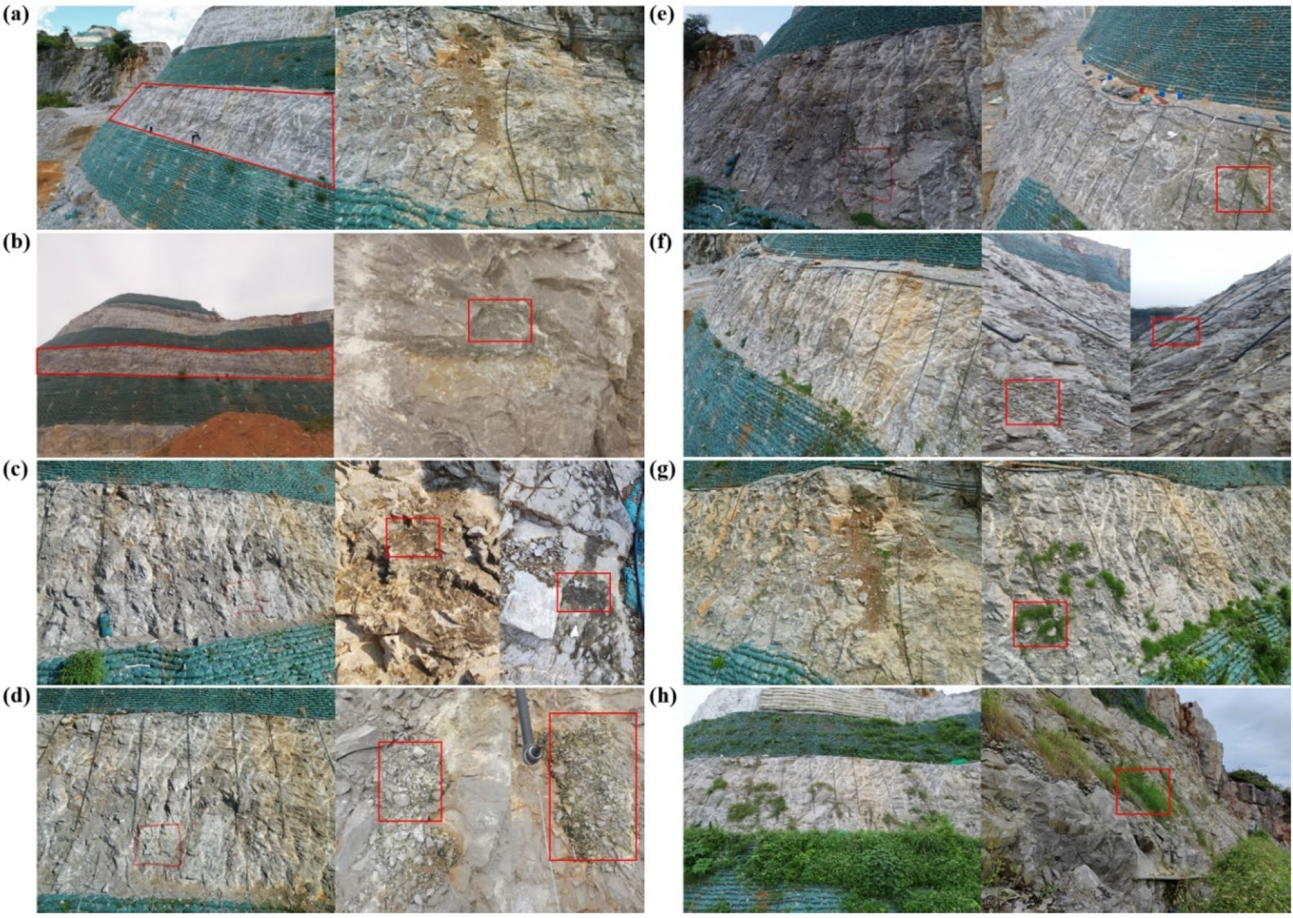
Site restoration record diagrams. (**a**) Experimental area after 15 days of restoration; (**b**) Experimental area after 30 days of restoration; (**c**) Experimental area after 60 days of restoration; (**d**) Experimental area after 75 days of restoration; (**e**) Experimental area after 90 days of restoration; (**f**) Experimental area after 180 days of restoration; (**g**) Experimental area after 210 days of restoration; (**h**) Experimental area after 240 days of restoration.

The microbial remediation approach employed in this study is underpinned by the theoretical framework of biological soil crusts^42,43^. It involves the binding of bacteria, fungi, algae, lichens, mosses, and their hyphae and secretions with soil particles to form a surface complex under specific climatic conditions. Biological crust organisms are pioneer species in the early stages of recovery in extremely degraded ecosystems and play a crucial role in promoting vegetation succession^44^. The characteristics of high and steep rocky slopes, with scarce soil and exposed bedrock, match the ecological restoration functions of biological crusts. Biological crusts intercept and store soil moisture through their unique vegetation morphology, providing conditions for the germination of other vegetation seeds. The biological crust, together with its associated enriched microbial community, can enhance soil hydrothermal regimes, stimulate nutrient cycling, and improve key soil physicochemical attributes^45^. Moreover, the proliferation of algal filaments, lichen rhizoids, and moss rhizines exerts mechanical disruption on rock surfaces and secretes carbonic anhydrase, thereby accelerating rock weathering and soil genesis in rocky environments^46^. The biological crusts covering the surface soil of slopes can enhance soil layer stability. Furthermore, biological crust cover increases surface roughness, significantly extending the initial runoff time on slopes, promoting soil moisture infiltration, thereby enhancing soil erosion resistance, and reducing soil and water loss^47^. To investigate the specific role of this compound microbial agents in the vegetation succession process on typical high and steep rocky slopes, this study continuously monitored the ecological restoration effects in the experimental area under different restoration periods with human intervention. The results are shown in Fig. 6.

Field monitoring results demonstrated a clear temporal sequence in the structural evolution of the rock surface and microbial community colonization. In the initial state of the experimental area (Fig. 1b,c), the rock walls exhibited minimal fissure development and no vegetation coverage. By day 30 of the restoration process, no significant macroscopic changes were observed, but slight color variations and the emergence of microbial (algal) spots were detected at the microscopic level. By day 60, some rubble accumulation became visible on the rock surface in the restoration area, accompanied by an increased density of microbial spots, indicating a more pronounced colonization by algae. By day 75, rubble accumulation had intensified, fissure expansion became evident, and conspicuous colonization by algae, lichens, and mosses was observed. These structures predominantly originated from depressions or fissures in the rock surface, which provided microhabitats favorable for microbial colonization. Compared with planar rock surfaces, these microtopographic depressions and fissures provide superior conditions for moisture retention and physical protection, thus fostering the proliferation of algae, lichens, and mosses^48^. At day 90, germination of herbaceous plant seeds was observed. However, due to declining temperatures, vegetation expansion remained limited. By the sixth month of restoration, after the removal of geotextiles and plastic film covers used during winter, a large portion of the biological crust structures persisted. In addition, the rock surface exhibited more pronounced signs of fragmentation, with accumulating rubble and rock mud creating a foundational substrate for further colonization by biological crust organisms.

After restoration for 210 days, the rock wall surface showed a significant increase in fissures, rubble, and grooves, and the surface structural strength changed. With the continued warming of the climate, vegetation growth gradually appeared. By the eighth month of restoration, vegetation establishment on rock surfaces had advanced markedly, with localized patches exhibiting more than 30% cover. Although this was less significant compared to the upper and lower vegetative bags, careful observation revealed that the vegetation grown in the vegetative bags were mostly single species and highly seasonal, whereas the vegetation growing in the experimental area, though fewer in number, were diverse and exhibited biodiversity. Additionally, the vegetation restoration effect in the vegetative bags below the experimental area was more pronounced than in the vegetative bags above the experimental area. This was due to the accumulation of some compound microbial agents from the spraying and infiltration of the microbial agents downward, further validating the superiority of the technology used in this study. Visual inspections suggested that these fissures were accompanied by dense growth of lichens and mosses, which likely facilitated the loosening and disintegration of surface rocks through mechanical penetration by rhizoids and hyphae, as well as the secretion of weathering-related enzymes. The interplay between these mechanical and biochemical processes likely played a substantial role in fissure formation and the detachment of rock fragments within the restoration zone.

In natural environments, it has been widely observed that reproductive bodies such as spores and stem-leaf fragments are transported to rock surfaces by wind or water flow, where they may develop into protonema under suitable conditions and eventually form gametophytes^49^. Spores require water absorption for swelling in the initial germination stage and light for energy and signal transduction during the extension stage. Similarly, stem-leaf fragments need water and light to maintain their life activities, making adequate water and nutrient supply crucial during their colonization^50^. Therefore, the reason this study achieved ecological restoration of high and steep rocky slopes using microbial spraying technology lies in the simultaneous introduction of functional microorganisms and provision of water for vegetation growth when spraying compound microbial agents. This process also enhanced soil fertility and optimized soil structure, thereby promoting vegetation growth in initially barren areas. Additionally, the dense rock structure has poor water retention. When the surface is smooth, fallen reproductive bodies struggle to colonize and develop. However, the larger spaces in rock depressions or cracks provide more room for settlement, reducing the impact of external forces and offering refuges for captured weathering products, soil, and dust deposits, creating favorable conditions for vegetation development^51^. These findings further support the hypothesis that the synergistic effects of mechanical pressure from biological structures and biochemical weathering by microbial communities played a central role in initiating rock fragmentation and promoting microhabitat formation. Such processes are fundamental to enabling vegetation establishment in initially barren, high-gradient rocky slope environments.

### Interactions among microbial communities, soil nutrients, and vegetation recovery

This study continuously monitored a representative high and steep rocky slope in the restoration zone and observed a coordinated evolution among microbial community structure, soil nutrient levels, and vegetation recovery. These results reveal potential causal relationships among the three components. Firstly, the diversity and dominant genera of microbial communities exhibited distinct successional trends during the restoration process. By day 15, the microbial community had already become dominated by genera such as *Ancylobacter* and *Thauera*, whose ecological functions involve nitrogen transformation, organic matter mineralization, and substrate degradation. These functions laid a microbial foundation for subsequent nutrient accumulation. As restoration progressed, the community structure stabilized, microbial metabolism accelerated, and the improvement of soil physicochemical properties was enhanced. Secondly, soil nutrient levels increased significantly during the restoration period, peaking around day 210. Specifically, soil organic matter content increased from 8.90 ± 0.75 g/kg on day 60 to 18.90 ± 1.39 g/kg on day 210—more than doubling. Available phosphorus rose markedly from below the detection limit (less than 2.0 mg/kg) to 11.10 ± 0.52 mg/kg. Both available potassium and total potassium remained at high levels, at 59.80 ± 8.46 mg/kg and 2980 ± 23.96 mg/kg, respectively. These improvements in nutrient content closely corresponded with microbial succession, suggesting that microbial activity significantly promoted the biological transformation of initial weathering products and the release of available nutrients. Furthermore, vegetation succession followed a clear progression. Germination of grass species was observed from day 90 onward, and by day 240, local areas of the experimental zone exhibited vegetation cover exceeding 30%, in stark contrast to the bare initial state. Early microbial activity enhanced soil fertility and facilitated plant germination, while vegetation growth in turn improved soil aggregation and microbial diversity through rhizosphere effects. This formed a positive feedback loop among microorganisms, soil, and vegetation.

In summary, the application of compound microbial agents accelerated the formation of biological crusts, promoted the colonization and metabolic activity of beneficial microorganisms, and enhanced soil nutrient accumulation and physical properties. These changes created essential conditions for subsequent vegetation establishment. This mechanism demonstrated significant effectiveness in the ecological restoration of high and steep rocky slopes and provides a scientific basis for the engineering application of microbial technologies in extremely barren environments.

## Conclusion

This project utilized compound microbial agents to restore typical high and steep rocky slopes, aiming to achieve ecological restoration of typical degraded areas through the metabolic activities of microorganisms and their interactions with soil and vegetation. The effectiveness of the compound microbial agents was evaluated based on soil nutrient content and vegetation growth in the experimental site. Additionally, 16 S rRNA gene high-throughput sequencing technology was used to analyze the microbial diversity in the experimental site to elucidate the role of functional microorganisms during the restoration process. The results showed that spraying compound microbial agents on the high and steep rocky slopes increased soil organic matter, available potassium, total carbon, and organic carbon content by 44.49%, 19.43%, 0.04%, and 0.03%, respectively. Total nitrogen and total phosphorus content increased by 5.3 mg/kg and 28 mg/kg, respectively, and available phosphorus content increased to 6.10 mg/kg. These improvements indicate that compound microbial agents not only supplemented nutrients but also enhanced soil fertility through sustained microbial activity, supporting vegetation colonization. Microbial community analysis revealed structural shifts toward beneficial taxa, including genera with confirmed environmental remediation potential, validating the ecological function of the microbial agents. Continuous monitoring of the ecological restoration of the high and steep rocky slopes showed that, with extended restoration time, there was a noticeable increase in fissures, rubble, and grooves on the rock wall surface, along with the emergence of algae, lichens and mosses, forming a foundation for the attachment of biological crusts. After a 240-day restoration period, over 30% of the rock surface was covered with vegetation in some areas. This study confirms that functional microorganisms can optimize soil structure and nutrient status, thereby accelerating vegetation establishment in barren rocky environments. The research outcomes provide a sustainable management method with both ecological and economic benefits for high and steep rocky slopes, offering a demonstration model and technical reference for the management of other similar sites.

## Data Availability

The datasets generated and/or analysed during the current study are available in the NCBI Sequence Read Archive (SRA) under the BioProject accession number PRJNA1307221 (https://www.ncbi.nlm.nih.gov/sra/PRJNA1307221).
